# Scrofula, Its Nature, Its Causes, Its Prevalence, and the Principles of Treatment

**Published:** 1846-11

**Authors:** 


### BUFFALO MEDICAL JOURNAL

AND

### MONTHLY REVIEW.

**VOL. 2.** NOVEMBER, 1846. **NO. 6.**

### ORIGINAL COMMUNICATIONS.

ART. I.—*Scrofula, its nature, its causes, its prevalence, and the principles of treatment.* By BENJAMIN PHILLIPS, F. R. S.

(Continued from page 282.)

It would to a considerable extent compensate for our ignorance of the pathology of Scrofula, if we could determine with positiveness and precision the agencies which produce it. We might then adopt measures, which, although empirical, and therefore less satisfactory than rational methods, would yet accomplish one of the great ends of our science, viz., prevention of disease. Besides, there would be good reason to anticipate that light shed on its etiology, might be reflected so as to lead to the discovery of its pathological character and relations. But when we inquire into the actual condition of science as regards the causes of scrofula, we find that here, as in other departments of the subject, there is almost an absolute deficiency of reliable, well established facts. As usual when a variety of suspected influences exist, to which, singly or combined, a disease is attributable, the number of doctrines nearly corresponds to the number of circumstances which can be imagined to sustain a causative relation to it. In treating of the subject, of course it is necessary to consider the grounds upon which the more prominent of these doctrines rest, in other words to inquire as to the importance which belongs to the several agencies which have been supposed to stand to the disease in the light of causes. This Mr. Phillips proceeds to do in the chapter entitled, *Causes of*

*Scrofula.* In giving a synopsis of the conclusions to which he arrives, we must here, as we have done with reference to the entire work, prepare our readers not to look for any very new and startling developments. We fear it must be acknowledged that his efforts to penetrate the obscurity which invests the subject, have not been attended with much success. Nevertheless, the data which he has accumulated are by no means useless. Many interesting and important considerations are connected with the statistical facts, by means of which the investigation is mostly prosecuted. He considers first the *Hereditary cause.* That the disease, to a greater or less extent, is due to causes which are inherited, is an opinion very generally entertained. M. Lugol, who is well known to have bestowed especial attention upon this subject, with large opportunities for investigation, maintains that this is the chief source whence it is derived and perpetuated. So exclusively does he refer it to hereditary causation, that supposing no evidences of scrofulous taint could be discovered by a close examination of the progenitors of a victim to the disease, he would assume that the child was begotten by adulterous intercourse, and that the paramour transmitted the seeds of the disease! We cannot readily cite a more striking illustration of the habit of inventing facts, to protect a hypothesis against those of an obvious character, which render it entirely untenable. Such a gratuitous supposition carries with it its own absurdity, and does not require any serious arguments for its disproval. On the other hand, able writers and observers have contended that, not only is this disease in no instance hereditary, but that no disease whatever is thus attributable; and, doubtless, they have been able to discover cogent arguments with which to maintain this doctrine. The questions, what amount of influence, if any, is derived from parents, and in what proportion of cases, if in any, is this cause operative, cannot be settled by past opinions, or by the belief of any individual, but are still open for discussion, and fresh investigations. The facts which were obtained on this subject, and the inferences fairly deducible therefrom, are contained in the following quotation:

"The means which I have taken to acquire accurate data as to the extent to which hereditary causes operate, in the propagation of scrofula, are the following. I examined myself, and procured to be examined by others, in the Metropolitan, the Factory, and in Rural Districts, upwards of 2000 families, each consisting of from three to five children, and living, as nearly as may be, under similar circumstances. In one portion of the cases, both parents were apparently free from scrofulous taint; in another portion, there was reason to think that both parents were tainted; in another, that the father was tainted; in another, the mother. The number of families examined was 2023, the number of children was 7587; and the number bearing such marks of Scrofula as I have already indicated, was 1738, or nearly 23 per cent. In 506 instances, derived from many localities, and under the most varied circumstances, both parents were apparently untainted, and their offspring amounted to 2021. Of these, 421, or something less than 21 per cent., presented marks of scrofula. In 276 instances, there was reason to think that both parents labored under scrofulous taint; their offspring amounted to 1092 children; of these, 271, or nearly 25 per cent., bore the ordinary marks of scrofula. In 589 instances, the father carried about him marks of having suffered from scrofula, whilst the mother was free from them; their children amounted to 2107, those having marks of scrofula, to 483, or nearly 23 per cent. In 652 instances, the mother bore upon her person the marks of scrofula, whilst the father did not; their children amounted to 2367, and of these, 563, or nearly 24 per cent., presented marks of scrofula.

It will be observed that although an hereditary influence must be admitted to be present, and is apparent in each class; yet at its maximum, the influence does not appear to be quite 4 per cent. It would seem that the influence of a scrofulous mother upon the offspring is greater than that of a scrofulous father.

I do not pretend to regard these results as an accurate representation of the influence of scrofula when existing in the parent to reproduce itself in the child. I would even admit, that as the cases were seen with many eyes, the data may be more defective than if they had been the result of one person's examination; but however defective they may be, they are the only approach I know of, to a reasonable amount of evidence, to enable us to judge how far it is probable that scrofula in the parent will reproduce itself in the child. And from that evidence, it would seem that in children subjected after birth to similar circumstances, the hereditary influence does not appear to be exerted beyond 4 per cent. This result is in opposition to two parties, one maintaining that the disease is always hereditary, and never acquired; the other, that no diseases are hereditary, but that they are always the result of circumstances which come into action after birth."

The inquiry will arise, may not the disease be connate, received by inheritance, but in another sense than is intended strictly to express by the term hereditary. If its development do not depend on a certain element, or virus, inherent in the constitution of the child, which was communicated by the act of procreation, or during gestation, may it not be due to an original predisposition originating anterior to birth? The latter is maintained by many who do not attach much importance to the former. Indeed, we apprehend that it is in this sense that the doctrine of hereditary transmission is generally received. It is not supposed that when a child of scrofulous parents dies of the disease, the result follows from hereditary influences alone, independently of those of an extrinsic character; but that in many instances in which scrofula does become developed under the immediate operation of external agencies, there is a congenital tendency which determines this, rather than other forms of disease. This inquiry may be extended by embracing, as a condition appertaining to parents, not only a scrofulous taint, but the existence of other forms of disease, more especially tubercular cachexy, and syphilis. May not the two latter forms of disease impose upon children a predisposition to scrofula? The affirmative to this question has been maintained by able writers. Sir James Clark, particularly, whose work on tubercular diseases is much esteemed, thinks that the children of tuberculous parents are especially prone to scrofula, and several authors of distinction have advocated the same with reference to syphilis. Mr. P. has not collected facts bearing directly on these questions. He adduces data which show that the proportion of weakly children who die tuberculous is vastly greater than the proportion of healthy children who fall victims to this disease; but, taking into consideration that in a prior portion of the work he has shown that there exists a kind of antagonism between mortality from scrofula and tuberculosis, we should say this fact militates against the supposition that general weakness constituted a predisposition to scrofula. The fact is, this point is left without adequate data for any inferences. The same is, also, true of syphilis. To collect statistics on this point to any considerable extent, would, obviously, be a matter of great difficulty, if not impossibility. From several considerations, however, he does not believe that there is any foundation for the suspicion that scrofula is developed in the offspring of those who have suffered from syphilis, as a consequence of the syphilitic taint. The considerations upon which this opinion is based, are briefly recapitulated in the following extract:

"From all these circumstances, I arrive at the conclusion, that scrofula and syphilis are independent the one of the other; that each has a character proper to itself, and that the same treatment is inapplicable to both diseases. For example, if scrofula be rife where syphilis is rare; if syphilis affects all periods of life while scrofula is more confined to a particular period; if hereditary syphilis be manifested at, or soon after birth, while scrofula unfrequently appears before the second or third year, if syphilis, whether inherited or acquired, be rarely cured spontaneously, and if, as every one knows to be the case, scrofula often disappears at the approach of puberty; if syphilis usually yield to mercurial treatment, while scrofula does not—should we not conclude that the one has no dependence on the other? Besides, if scrofula were derived from syphilis, it surely would not be rare to find it in those children who have resulted from infected parents, and to whom syphilis has been hereditarily transmitted. But how frequently do we see syphilis thus communicated, and how rarely, at the same time, do we find the child presenting a scrofulous taint."

Various other circumstances connected with parentage and procreation have been imagined to be concerned in the production of scrofula. Among these are the old age of parents, the occurrence of conception during menstruation, "marrying in-and-in." All these are mere assumptions, having no support from a sufficient number of well attested facts.

We pass now to speak of causes operating *after* birth. And first, will *the milk of a scrofulous nurse tend to produce scrofula in the child?* It is a common impression that the disease does sometimes originate, or, at least, the elements of it are received in this way. It is a point of much practical importance to decide how much value is to be placed on this impression. The author does not submit any statistics, but he states that he knows "of no single well observed fact on record in support of the opinion." Menstruation has been very generally supposed to impair the qualities of the milk of nurses. It does not appear, however, from an analysis of the secretion, that any essential change takes place. M. Raciborski has made some careful observations upon milk under these circumstances, and the only change detected was a diminished proportion of cream. There would seem, therefore, to be no necessity for rejecting a nurse for this reason, as authorities usually recommend.

This fact, which is incidentally introduced, we quote for its practical importance. *Contagion* has been supposed by some to exist as a causative principle in perpetuating scrofula. At the present time, however, there are but few, if any, who hold to this idea. It is a sufficient disproval of it that inoculation of scrofulous matter has been repeatedly and faithfully practiced without the production of any scrofulous infection. Experiments for this end have been made, not alone on inferior animals, but on the human subject. There is as little foundation for the opinion, which has also been entertained, that the introduction of pus arising from other diseases is capable of producing scrofula.

But there must be some agencies, external to the body, which are instrumental in determining the disease. At least, this presumption is rendered more rational by the fact that there exists so little proof of the validity of those already mentioned. Food, atmosphere, climate, occupation, constitute classes of external influences in which, separately or collectively, the causes of the disease may reside. The relative importance of these causes is now to be considered. To bring statistical data to bear on these circumstances, individually, is not an easy task, because we cannot readily collect sufficient numbers of cases in which uniform conditions are observed. It is difficult to study the influence of each separately, because they are always of necessity more or less combined, and, hence, we are unable to determine how much is due to one element taken singly, or how much to the combined effect. These difficulties apply to direct statistical results; still, indirectly, some numerical facts may have an important application.

As preliminary to the investigation of the common agencies by which the body is surrounded, the interesting fact is to be stated, that by reference to the tables exhibiting the general mortality in different parts of Great Britain, the following law is determinable: in the localities presenting the greatest mortality from all diseases, the deaths from scrofula are in the smallest proportion. A similar law, as previously remarked, relates to mortality from Phthisis. Deaths from scrofula are inversely in proportion to the prevalence of tubercular disease of the lungs. From the first of these laws Mr. P. is led to deduce that many of the causes which tend to the destruction of life, when they act with greatest energy, do not develop scrofula, but other diseases productive of a greater mortality; but acting with less energy, they may lead to the development of scrofula. The reader will probably see room for skepticism as to the propriety of giving to this inference any more weight than may be attached to a hypothesis or suspicion.

On the subject of food the author submits a large collection of statistical data. We should be glad to give them in substance, but we find it will occupy too much space, and we must therefore content ourselves with stating, that by a comparison of the proportionate number of deaths from scrofula, and its prevalence in different countries, public institutions in different portions of the same country, &c., where the supply of wholesome food is most abundant, and the ability to obtain it more generally diffused, with localities and conditions where it is otherwise, he shows a striking disparity, the former preponderating very considerably over the latter. On this topic he has accumulated a large number of data, gathered from a great number of sources, which the critical reader, fond of this method of investigation, will do well to examine. The following is the conclusion at which he arrives:

"I think, then, it has now been shown that in Great Britain, scroula is least prevalent where children and others are best fed, and although I by no means assume that the immunity is entirely owing to better feeding—because where much attention is bestowed upon the food, it is hardly likely that other means of maintaining health will be neglected; yet I would submit as a fair deduction from the foregoing evidence that food exercises a more important influence than any other agent in the production of scrofula."

With reference to the influences of different kinds of food, without adducing a series of comparative observations, he expresses the following opinion:

"That those who live almost exclusively on vegetable food in this country are less robust, and exhibit a greater tendency to scrofula, than those who subsist on an admixture of animal and vegetable food, is, I think, true. Our own rural population, as well as that of Scotland and Ireland, bear out the assertion. But, although it has been shown, that insufficient and improper food, however associated, may lay a foundation for that disease, we have in truth, no conclusive proof that any particular article of food directly tends to the production of scrofula."

*Of Atmosphere.*—In our common ideas of the causation of scrofula, an impure atmosphere, by over crowding, and other causes, is supposed to exert an influence scarcely less potent than insufficient or unwholesome food. By some authors the disease is attributed exclusively to this source. Such was the opinion of Baudelocque, and also Richerand. To test the amount of agency exerted in this way statistically, it seems fair to contrast the mortality from scrofula in metropolitan and rural districts. This is liable to the objection that the difference in atmosphere is not the only point of distinction between the influences of town and country. Still, it is one of the prominent points of contrast, and it would reasonably be expected if this cause were in an especial manner operative in the production of the disease, it would, at least, be found that the mortality from scrofula is greater proportionably in the former. It happens, however, from the data collected by Mr. Phillips, that precisely the reverse is true — a fact which must excite some surprise. In large towns, not including London, of 32,670 children examined, 18½ per cent. were returned as scrofulous; while in rural districts, of 20,540 examined, 29 per cent. were returned as scrofulous! Taking the report of the Registrar General for a period of four years in a district comprising towns having a population of 3,759,186 souls, the deaths from scrofula were 758; and in a rural district having a population of 3,440,501 souls, the deaths amounted to 1333. In other words, the proportion of deaths from scrofula to 1,000,000 living, was in the town districts 50 per annum, and in the country districts 97! Again comparing one portion of the metropolis (London) with another, the total deaths from scrofula, are as 5-6 to 100,000 living. Whilst in the most densely crowded districts the is as 5-1 to 100,000! So in the several low, dense and poor districts, in which the returns were examined, the deaths were as 1 to 478, while in districts of an opposite description they were as 1 to 490! The fact, also, which has been already stated, that boys are more liable to the disease than girls, militates strongly against the operation of this cause, since, it must be admitted, that girls are much more exposed to the deleterious effects of crowded rooms, and the absence of out-door exercises. These results will, we think, be deemed by our readers highly interesting. As regards the importance to be attached to them in their application to the subject under consideration, we do not feel prepared to express an opinion. Should they be confirmed by more extended, and by repeated statistical data, they must certainly be deemed to controvert views which have been very generally entertained.

*On Climate.*—The data collected on this point do not lead to results by which any of the ordinary elements of climate are shown to be instrumental in the production of scrofula. In a comparison of countries in which humidity is a principal climatic element, the inference is that a small influence is exerted in the production of the disease. The data collected are, however, as the author states, inadequate to a conclusive deduction. The difficulty which attaches to all departments of the investigations are particularly applicable to climate. Climate embraces several ordinary, as well as extraordinary elements: how can investigations be conducted so as to give results showing the influence of each separately? It is difficult, if not impossible to accomplish the object, and many may be disposed to attach little or no value to any data which may be collected, in authorising deductions as to peculiar climatic influences. At most the results are to be regarded as partial evidence of the correctness of the deductions which seem to follow.

*Temperature* is one of the most prominent features of climate, and if it be involved greatly in the creation of the disease, it would seem that statistics would determine the fact. Mr. Phillips attaches considerable value to the returns from the British Army, as bearing on this topic. They furnish statistics of diseases in different climates, and under a great similarity of condition as respects food, clothing, &c.; and they are spread over a great number of years. "It does not appear (from these returns) that either a high or a low, an equable or a variable temperature, exercises any uniform or evident influence over the occurrence of scrofula in the British soldier." Scrofula, however, is much more prevalent is some countries than in others. In India it appears to prevail very extensively among the natives, but not among the European residents. At St. Petersburg, Moscow, and in Iceland, there is less of the disease than at Lisbon, Amsterdam, Berlin, or Calcutta. Certain indigenous influences are, of course, instrumental in such variations, but we are not warranted in referring them to temperature, nor to any of the ordinary climatic elements.

We come lastly to *Occupation*. And here the results of Mr. Phillips' investigations are quite at variance with current impressions. It has been supposed that the circumstances attendant upon factory labor tend particularly to promote the disease; but it appears from the data here presented, that the converse of this is true — factory labor seems to involve, to some extent, an exemption from Scrofula! Comparing the returns from several factory towns, with those from towns with a population not employed in factory labor, the ratio of the prevalence of the disease is decidedly greater in the latter! In confirmation of this result, Mr. P. adduces testimony of several medical men residing in factory towns, or in the neighborhood of extensive factories, not only to the infrequency of this disease, but of others tending to the destruction of life. We give in the author's words his conclusions on this subject:

"The evidence I have collected upon the question of the influence of factory labor upon human life, has produced on my own mind a strong conviction, that occupation in factories, though in many respects less to be desired than occupation in the open air, is yet accompanied by so many counteracting circumstances, that the evils which may be inseparable from that occupation are mitigated if not counteracted by the increased means of procuring the necessaries of life which it affords. The evils of factory towns do not consist in factory employment, but in the agencies which are commonly found in action in such towns."

On reviewing the several causes which have been mentioned, it appears that we are not authorised by statistical data to consider any single one as standing to the disease in an exclusively causative relation. Nor does it appear that to either alone is the disease due in a large proportion of cases. Those which are concerned in its production more than any class of causes relate to the functions of nutrition. This is shown by data which have reference to the influence of food, and perhaps we are justified in concluding, from the little or no appreciable influence apparently exerted by other causes, that this is operative to a much greater extent than the positive results of statistical data will sanction. In so far as external causes are concerned, it seems to us that the subject thus resolves itself:— Either the various causes tending to impair nutrition are the chief agents determining the disease, under the great variety of conditions and circumstances which attend it; or, it is produced by the combined action of several causes in a large proportion of cases; or, again, the etiology involves some specific agency, or agencies, which are yet to be discovered.

We pass now to the concluding portion of the work, which discusses the prevention, and the principles of treatment. This we shall dismiss with brief notice, not because it does not contain much that is interesting and valuable, but it cannot be said to embrace any new principles of therapeutics, and does not well admit of analysis. Under the head of preventive management, the general principles of dietetics and regimen for infancy and childhood are clearly, and, as it seems to us, judiciously presented. The author is opposed to the custom of tasking the intellectual powers prematurely, and subjecting children to the confinement and depressing discipline of the school room, to the extent frequently practiced by over ambitious parents and instructors. There cannot be a doubt that this hot-house system of forcing intellectual growth, is attended with a great sacrifice of life and health. For the rest we must refer our readers to the work.

Under the head of curative treatment, he considers briefly the several agents which enjoy more or less confidence of the profession.

*First, of Mercury.* He denies that upon this agent any reliance is to be placed for the cure of scrofula, but does not doubt that in many cases it proves of use. The form which he deems to answer best is the *Bichloride* in very minute doses, for instance, a twentieth of a grain twice a day, with the syrup of sarsaparilla. In this mode it frequently is equal in efficacy to any other remedy.

*Second, of Iodine.* The observations of Coindet and Lugol have given to this remedy almost the character of a specific in scrofula. Yet, when judged by the reports of Lugol himself, it does, by no means, possess absolute power over the disease. While he vaunts it as almost certainly reliable, his cases show that out of 109 cases only 39 were discharged cured! Such are inconsistences and self-deceptions of an extravagant enthusiasm! Mr. Phillips does not estimate the powers of the iodine so highly as many others, but that it exerts a real efficacy in many cases he does not doubt. Often, however, he thinks the benefit attributed to it is due to general hygienic influences associated with it. He prefers the Iodide of Potassium to the Iodine alone. He has also found the Iodide of Iron in many cases especially useful. He thinks after being continued for two or three weeks it should be discontinued for a while, and again resumed, if necessary.

*Third, of Barium.* He has found this useful. He gives it in solution i. gr. to an ounce of distilled water. Of this he commences with half an ounce twice daily, and on no account exceeds three grains daily. *Hydrochlorate of Lime.* He has never seen a case in which this remedy exerted any manifest discutient powers.

*Fourth, of Alkalies.* These have long enjoyed popularity. We may add that their popularity is much greater in England than in this country. He is satisfied that in many cases they exert a salutary effect, but not to the extent claimed by those who have most highly recommended it.

*Fifth, of Burnt Sponge.* This has no advantage over preparations of Iodine.

*Sixth, of Cod-liver Oil.* This is just now the favorite remedy in England. We do not hear much of its use in this country. Some traces of Iodine and Bromine have been discovered in it. He has used it to a considerable extent, and has seen every form of scrofulous disease improve under its use. He attributes its efficacy in a measure to the animal oil, irrespective of its other qualities.

*Seventh, Sea-side Residence.* That this is often useful is evident. In the absence of proof, however, that people residing at the sea-side are more free from scrofula than those who live in-land, he does not attribute the benefit to aught derived from contiguity to the sea, but to the various influences attendant on change of residence.

On the subject of mineral waters he adduces the opinions of various writers. He does attribute to them any direct virtues, but explains their apparent efficacy in the same way as sea-side residence.

Finally, he closes with some general suggestions which are well worthy of attention, but do not admit of condensation.

At the risk of extending this article (perhaps already too long) beyond the limits, which, in the judgements of our readers, it should occupy, we will give an extract from his concluding chapter. The subject is one of interest, but we will not presume upon the patience of our readers by any remarks of ours.

"And now that the task I have undertaken has been brought to a conclusion, I would shortly allude to a question which has forced itself upon my attention, from the beginning to the end of the inquiries in which I have been engaged, namely: What is the influence of civilization upon the physical vigor of a people? There can be no question, I apprehend, that it tends to the preservation of weak and ailing members of the community, who would not be reared under less favored circumstances, but who are thus made the parent stock from which families take root.

Although the materials which we possess, do not enable me to offer any positive evidence on that question; and although some of the results which have been indicated, in the preceding pages, would seem, at first sight, to favor an opposite conclusion, I entertain a strong belief that the races which now occupy the more enlightened countries of Europe, have physically degenerated, and that civilization, including as one of its agents, improved medical science, has contributed to that result. Under a less advanced civilization, the weaker and least vigorous members of a community die off, in early life, and the more robust and healthy members alone are reared. In more advanced stages of social progress, however, the case is very different. In those states many weakly subjects are brought to maturity by intelligent care. And yet science, which has contributed to preserve the lives of the weak and ailing, can rarely give them vigorous health. When reared, they contribute to the spread of population, and not unfrequently give birth to children equally weak and ailing with themselves, if indeed their progeny do not evince even greater tendencies, to particular diseases, than the sickly parent.

Injurious influences such as these, it is true, are modified by various compensating agencies, such as intermarriage with persons of a robust and hardy frame, or other causes, and therefore operate slowly and imperceptibly, and can only be estimated by comparing the physical qualities of the same races at different and distant periods of their history. The rise and fall of nations testify, however, as distinctly to physical decline as to moral and intellectual degeneracy, and however greatly the progress of science and the spread of knowledge may increase the resources of society and the power of communities, they do not always secure to the individual man a physical vigor like that which in the early history of nations, elevated so many men above the general level. And if it be said, that such a conclusion is humiliating to the pride of science, when engaged in the noblest of all her missions, as the handmaid of suffering humanity, the reflection will not be a without a wholesome influence, since it tends to humble our pride and impress us with the salutary truth, that man's power to remove suffering, or to mitigate evil, is confined within very narrow limits."

We have given this work so extended an analysis, in the first place, because the subject is one of great interest and importance; in the second place, it is altogether the most elaborate monograph on this, and perhaps it may be added, or any other subject of medical literature. It is true that the conclusions at which the author arrives are negative rather than positive. In other words, he has not succeeded in elucidating the pathology, or the etiology of the disease, nor does he introduce any new principles of treatment. But we have here presented materials by which we can certainly, better than before, estimate the importance of many of the ideas and doctrines which have been, from time immemorial, connected with the subject. He shows us that much of what we have flattered ourselves was real knowledge, after all, is but hypothetical, and must probably be abandoned. We think that in this he has rendered valuable service to science; nor should we refuse to render our thanks for it, although it would have been more agreable had he established new views of the pathology, etiology and treatment of scrofula. We had designed to have reserved space for some remarks on the subject of statistics, as applicable to the present disease, and to medical investigations in general. This article, however, is already so much extended that we must forego this intention. We would simply say, that there is perhaps a prevailing tendency to carry this method to an extreme, and to rely upon it too exclusively. While we believe that it will prove a powerful instrument for the investigation of truth, we do not believe it is to supersede other methods. Care must be taken not to be misled by the attractive idea of inflexible certainty and precision with which mathematical results are invested. Figures do not, in all their applications, always speak the truth. There are, in fact, abundant liabilities to error in their application to medical subjects, which should lead us to exercise caution in the reception of doctrines or principles based upon statistics alone. We are not prepared to say how appositely these remarks are in connection with the work which we have passed in review. We do not, however, make them because we consider them especially applicable here, but because the occasion suggests them.

We have said nothing of the literary merits of Mr. Phillips' work. The subjects are well arranged, the style is simple and lucid, and, altogether, it possesses many readable attractions. The author, also, evinces throughout a candor and truth-seeking spirit, which secures the confidence of the reader. The general mechanical appearance is excellent, but we cannot refrain from alluding to the many typographical errors which are to be found on almost every page. Should the work attain to a second edition, we hope the publishers will secure in its behalf the services of a more faithful proof reader.

